# Arp2/3-mediated F-actin formation controls regulated exocytosis *in vivo*

**DOI:** 10.1038/ncomms10098

**Published:** 2015-12-07

**Authors:** Duy T. Tran, Andrius Masedunskas, Roberto Weigert, Kelly G. Ten Hagen

**Affiliations:** 1 Developmental Glycobiology Section, NIDCR, National Institutes of Health, Building 30, Room 426, 30 Convent Drive, MSC 4370, Bethesda, Maryland 20892, USA; 2 Intracellular Membrane Trafficking Section, NIDCR, National Institutes of Health, 30 Convent Drive, Bethesda, Maryland 20892, USA; 3 Oncology Research Unit, School of Medical Sciences, University of New South Wales, Wallace Wurth Building East (C27), Room 227, Sydney, New South Wales 2052, Australia

## Abstract

The actin cytoskeleton plays crucial roles in many cellular processes, including regulated secretion. However, the mechanisms controlling F-actin dynamics in this process are largely unknown. Through 3D time-lapse imaging in a secreting organ, we show that F-actin is actively disassembled along the apical plasma membrane at the site of secretory vesicle fusion and re-assembled directionally on vesicle membranes. Moreover, we show that fusion pore formation and PIP_2_ redistribution precedes actin and myosin recruitment to secretory vesicle membranes. Finally, we show essential roles for the branched actin nucleators Arp2/3- and WASp in the process of secretory cargo expulsion and integration of vesicular membranes with the apical plasma membrane. Our results highlight previously unknown roles for branched actin in exocytosis and provide a genetically tractable system to image the temporal and spatial dynamics of polarized secretion *in vivo*.

## Abstract (web summary)

The cytoskeleton plays a crucial role in secretion. Here Tran *et al.* demonstrate that cortical actin is rearranged at the site of vesicle fusion and recruited to fused secretory granules in Drosophila salivary glands, and show that branched actin nucleators are required for cargo expulsion.

## Graphical abstract

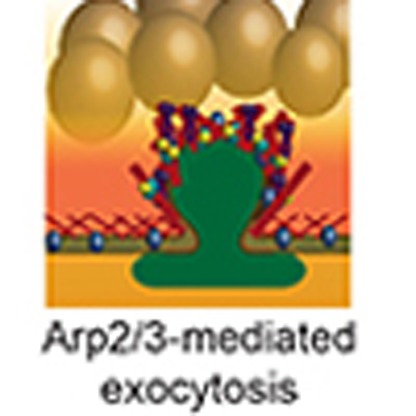

Regulated exocytosis is a fundamental cellular process that involves the release of stored secretory cargo from membranous vesicles into the extracellular space. This type of secretion involves the formation of secretory vesicles and their subsequent docking and fusion with the plasma membrane (PM) in response to an external stimulus. Various tissues have different modes of vesicle formation and release, likely due to the different physical and functional properties of the secreted cargo as well as the size/geometry of the secretory vesicles. Regulated secretion is used by specialized tissues to produce and secrete bioactive molecules that mediate diverse functions including neurotransmission, immunity, reproduction and digestion (reviewed in refs 1, 2, 3).

Recent studies have highlighted essential, yet often disparate roles for the actin cytoskeleton in regulated exocytosis and in compensatory endocytosis. For example, actin along the cortex of cells acts as a physical barrier to prevent premature fusion of granules with the PM^4^,^5^,^6^. In Xenopus eggs, where secretory vesicles never integrate into the PM but are maintained as shells, actin and myosin recruitment enable vesicles to be retrieved via compensatory endocytosis^7^,^8^,^9^,^10^. Other studies have demonstrated that F-actin is required for proper vesicle compression and expulsion of cargo^11^,^12^,^13^,^14^ and to regulate the expansion of the fusion pore^15^. In addition, intravital imaging in rodent salivary glands (SGs) has shown that F-actin is required to regulate both the stabilization and collapse of secretory vesicles after fusion with the PM *in vivo*^16^. However, the molecular machinery controlling the formation of specific F-actin structures and the spatial kinetics of actin assembly in the process of cargo expulsion and membrane integration has not been fully defined^3^.

Actin exists as monomeric units (G-actin) and in a variety of filaments that can be linear, branched or bundled (F-actin). Linear actin filaments are initiated by the formin-family of actin nucleators/elongation factors that are activated by the concerted action of small Rho-GTPases and phosphoinositides^17^,^18^,^19^. Rho and formin influence actin coat formation on lamellar bodies during surfactant secretion^12^,^13^. Branched actin filaments, which are essential for cell polarity and migration^20^,^21^, are formed through the coordinated action of the Actin-related protein 2/Actin-related protein 3 (Arp2/3) complex and a variety of nucleation-promoting factors (NPFs), such as WASp, Wash, Whamy and suppressor of cAR (SCAR)^20^,^22^,^23^,^24^. Arp2/3 present on linear actin filaments is subsequently activated by NPFs, which bind Arp2/3 and induce a structural change. Branched actin is then formed at the site of Arp2/3 attachment at a 70° angle from the existing actin filament^25^,^26^. Actin nucleators influence glucose-induced biphasic insulin secretion, but their specific effects on actin remodelling in this process are unclear^27^. The role of branched actin assembly and structure is less well understood in other forms of secretion that involve large secretory vesicles containing bulky cargo^3^.

The factors that govern regulated secretion have been challenging to decipher, as many mammalian cells lose polarity and secretory capacity once excised and cultured *ex vivo*^28^. Additionally, in many systems, the small size of vesicular structures and the lack of markers for cargo impede the ability to image actin dynamics, secretory vesicles and secretion with sufficient resolution. Furthermore, limited genetic tools in many systems have restricted the ability to interrogate the function of specific genes involved in actin formation *in vivo*.

To investigate the role of actin dynamics and branched actin in secretion, we performed high-resolution, real-time imaging at single-granule resolution in *Drosophila* SGs, which are amenable to genetic manipulation and contain large secretory granules (3-8 μM in diameter) filled with high-molecular-weight cargo. Using *Drosophila* lines expressing fluorescently-labelled molecules, we are able to directly image secretory vesicle cargo, apical membrane dynamics and actin and myosin recruitment in real time to define the temporal and spatial sequence of events occurring during regulated exocytosis. By performing 4-dimensional (4D) imaging of actin dynamics during secretion, we show rearrangement of cortical actin at the point of vesicle fusion followed by coordinated recruitment of actin to the fused vesicle from the PM. We further demonstrate that fusion pore formation and redistribution of the phosphoinositide, PIP_2_, occurs before actin recruitment. By imaging cargo directly, we show that fusion pore formation and cargo secretion are temporally distinct events, with the latter being dependent on actin for completion. Finally, using *in vivo* RNA interference (RNAi), we identify essential roles for the branched actin nucleators Arp2, Arp3 and their activator, WASp, in mediating cargo expulsion and secretory granule membrane integration with the PM. This study demonstrates a previously unknown role for branched actin nucleators in exocytosis and offers a high-resolution, genetically tractable platform for interrogating the role of any factor involved in actin dynamics and regulated secretion in a living organ in real time.

## Results

### Spatial and temporal dynamics of regulated secretion

**Figure 1:**
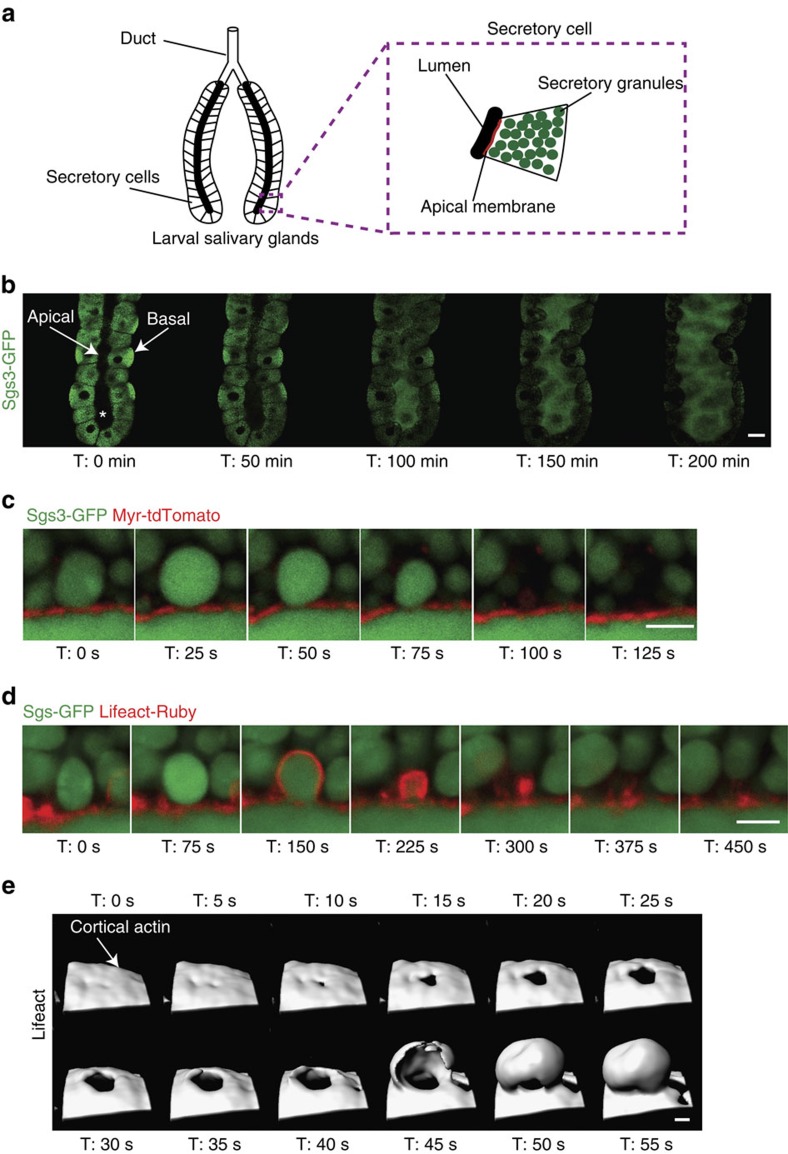
Real-time imaging of actin dynamics during regulated exocytosis. (**a**) Schematic diagram of third instar larval salivary glands (SGs). Inset depicts a single secretory cell. (**b**) Third instar larval SGs expressing Sgs3-GFP cargo (green) were imaged in real time after induction of secretion by the addition of 20E. Sgs3-GFP cargo in secretory vesicles is secreted into the lumen over time, leading to lumenal expansion. The distal tip of the gland is at the bottom of the image. Apical and basal surfaces are labelled. The SG lumen is denoted by a *. A representative time series from four independent experiments is shown. Scale bar, 50 μm. (**c**) Individual granules from SGs expressing Sgs3-GFP (green) and Myr-tdTomato (red; an apical PM marker) were imaged to show vesicle fusion with the PM and deformation of the PM at the site of fusion. After cargo expulsion, integration of granular membrane with the PM is seen. A representative time series from six independent experiments is shown. Scale bar, 5 μm. (**d**) Granule fusion in glands expressing Sgs3-GFP (green) and Lifeact-Ruby (red) shows actin recruitment to the fusing secretory granule, followed by expulsion of green secretory cargo into the lumen and integration of the granule into the PM. A representative time series from six independent experiments is shown. Scale bar, 5 μm. (**e**) Surface reconstruction of 4D Lifeact-GFP (white) imaging during granule secretion showing actin clearance at the apical PM followed by actin recruitment from the PM to the secretory vesicle. A representative time series from nine granule reconstructions over three independent experiments is shown. Scale bar, 3 μm. Complete movies for each time series can be found in the Supplementary Movie section.

The *Drosophila* larval salivary system consists of two glands comprised of secretory epithelial cells that connect to a common duct (Fig. 1a). Secretory cells are filled with large secretory granules (3–8 μM in diameter) containing high molecular weight, highly glycosylated mucins (salivary gland secretion proteins or sgs proteins)^29^,^30^. Regulated secretion in *Drosophila* SGs begins with a small pulse of the steroid hormone 20-hydroxyecdysone (20E) during early third instar larval stage. This stimulus induces the expression of the *sgs* genes that encode the cargo that will be packaged into secretory vesicles; then, a second pulse of 20E during late third instar instructs these newly formed secretory granules to fuse with the apical PM to begin secretion^31^,^32^,^33^. To image secretion events in real time, we took advantage of *Drosophila* lines expressing fluorescent markers for secretory cargo (Sgs3-GFP)^32^, F-actin (Lifeact-Ruby, Lifeact-RFP or Lifeact-GFP)^34^,^35^ and the apical membrane (Myr-tdTomato)^36^. SGs from Sgs3-GFP-expressing larvae were harvested during late third instar and cultured *ex vivo*. Cultured SGs maintain their polarity and respond to the exogenous addition of 20E, which triggers fusion of the secretory vesicles with the apical PM and release of their contents into the lumen (Fig. 1b). As secretion proceeds, the diameter of the lumen expands as it fills with the Sgs3-GFP cargo (Fig. 1b and Supplementary Movie 1), similar to what is seen *in vivo* (Supplementary Fig. 1a). (Real-time videos of all still images shown throughout this paper are in the Supplementary Movies section.) Imaging the dynamics of individual secretory granules using *Drosophila* lines expressing Sgs3-GFP cargo and the apical PM marker Myr-tdTomato revealed apical membrane deformations at the point of vesicle fusion (Fig. 1c). In addition, individual secretory vesicles undergo a slight expansion after fusion with the apical PM, likely due to hydration-related expansion of the mucin cargo. Real-time imaging of secretory cargo revealed that secretion proceeds with complete expulsion of cargo followed by integration of the vesicle membranes with the PM (Fig. 1c and Supplementary Movie 2).

We next imaged the dynamics of actin recruitment to individual secretory granules using *Drosophila* lines expressing both Sgs3-GFP and Lifeact-Ruby. Secretory vesicles in proximity to the apical membrane are rapidly coated with actin, which precedes vesicle compression and membrane integration (Fig. 1d and Supplementary Movie 3). To image actin recruitment around the entirety of the vesicle, we performed three-dimensional imaging in real time (4D imaging) in *Drosophila* lines expressing Lifeact-GFP. Interestingly, 4D reconstruction of actin dynamics showed that cortical actin present along the apical membrane is cleared or disrupted at the site of vesicle fusion, forming a small hole that continues to expand in size (Fig. 1e and Supplementary Fig. 1b,c and Supplementary Movies 4, and 5). This actin clearance is followed by actin recruitment to the secretory vesicle, originating at the PM (Fig. 1e and Supplementary Fig. 1b, c and Supplementary Movie 4, and 5). These 4D reconstructions demonstrate dynamic actin clearance at the PM and subsequent directional actin recruitment to the secretory vesicle from the PM during SG secretion. Taken together, we show secretion of mucinous cargo at single granule resolution that involves dynamic rearrangement of both the cortical actin and the apical membrane at the site of secretory vesicle fusion. This process culminates in complete expulsion of cargo and integration of the granular membrane with the PM.

**Figure 2:**
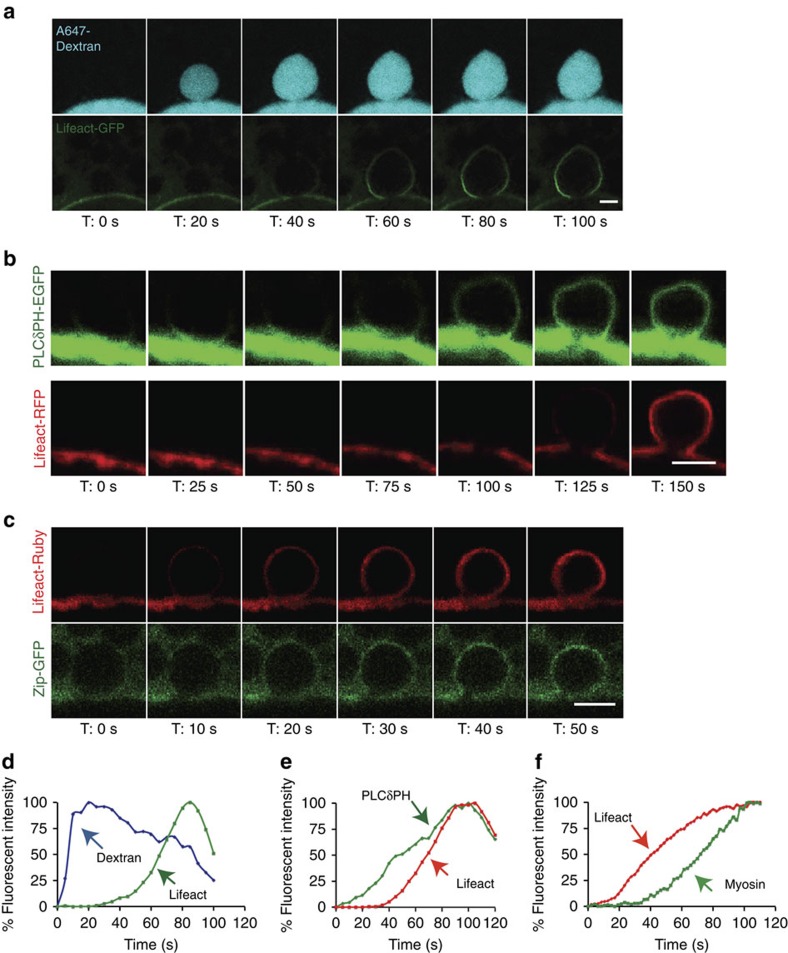
Fusion pore formation and membrane mixing occur before actin and myosin recruitment on individual secretory granules. (**a**) The lumen of glands expressing Lifeact-GFP (green) was infused with A647-dextran (cyan) to image fusion pore formation between the granule and the apical PM. Real-time imaging shows fusion pore formation (via diffusion of dextran from the SG lumen into the granules) before actin recruitment to the granule. A representative time series from five independent experiments is shown. Scale bar, 5 μm. (**b**) SGs expressing the PIP_2_ reporter, PLCδPH-EGFP (green) and Lifeact-RFP (red) were imaged in real time to demonstrate that diffusion of PIP_2_ from the apical membrane to the fused vesicle membrane occurs before actin recruitment. A representative time series from four independent experiments is shown. Scale bar, 5 μm. (**c**) Glands expressing Lifeact-Ruby (red) and Zip-GFP (green; non-muscle myosin II) were imaged in real time to show that actin recruitment to fused granules occurs before myosin recruitment. A representative time series from eight independent experiments is shown. Scale bar, 5 μm. (**d**) Quantification of Lifeact recruitment relative to time of dextran entry into fused granules. (**e**) Quantification of PLCδPH appearance relative to Lifeact recruitment to fused granules. (**f**) Quantification of Lifeact recruitment relative to myosin recruitment to fused granules. Representative graphs plotting percent fluorescent intensity of each marker as a function of time for individual secretory events are shown. Quantification was performed on 11 individual secretory events over two independent experiments, 12 individual secretory events over two independent experiments and 11 individual secretory events over four independent experiments, respectively. Additional graphs are shown in Supplementary Figs 2a, b and 3a. Complete movies for each time series can be found in the Supplementary Movie section.

We next addressed the temporal order of membrane fusion (fusion pore formation) and actin recruitment to the secretory vesicles. By infusing a small molecular weight (MW) fluorescent dextran (A647-10 kDa dextran, stoke radius ∼2 nm (ref. 15)) into the lumens of SGs expressing Lifeact-GFP, we demonstrate that the fusion pore between the vesicle and the PMs forms before F-actin recruitment to the secretory vesicle (Fig. 2a, d and Supplementary Fig. 2a). Indeed, dextran diffusion from the SG lumen into the vesicle was detected before Lifeact-GFP (F-actin) on vesicle membranes (Supplementary Movie 6). The average time difference in the detection of dextran relative to actin was 57±18 s (*n*=11; ± refers to standard deviation). In addition, we did not see any evidence for compound exocytosis (for example, the sequential fusion of vesicles with one another at the PM), consistent with what was previously reported in mammalian SGs^16^.

Membrane mixing and the redistribution of phosphoinositides are known to regulate actin polymerization and membrane fusion events in other systems^37^. In particular, the membrane phosphoinositide PI(4, 5)P_2_ (PIP_2_) is known to stimulate actin polymerization by regulating the activity of actin nucleators^38^,^39^. Therefore, we next examined whether redistribution of PIP_2_ occurs during SG secretion by using *Drosophila* lines expressing both the PIP_2_ reporter PLCδ-PH-EGFP^40^ and Lifeact-RFP. Indeed, evidence for membrane mixing via PIP_2_ movement from the apical PM to secretory vesicle membranes is seen (Fig. 2b,e and Supplementary Movie 7). Interestingly, PIP_2_ recruitment precedes F-actin recruitment (by 51±19 s (*n*=12)), consistent with a role for PIP_2_ in F-actin formation along secretory vesicle membranes (Fig. 2b,e and Supplementary Fig. 2b and Supplementary Movie 7).

In rodent exocrine glands, it was shown that the actin motor non-muscle myosin II is recruited onto the secretory granules, but the temporal sequence of events is not known^16^. To determine the kinetics of myosin recruitment relative to actin recruitment, we performed time-lapse imaging in flies co-expressing the myosin marker non-muscle myosin II (Zip-GFP)^41^,^42^ and Lifeact-Ruby. As shown in Fig. 2c,f and Supplementary Fig. 3a and Supplementary Movie 8, myosin is detected on fused vesicles after F-actin. The average time difference in the detection of actin relative to myosin was 25±13 s (*n*=11). Altogether, our data show that secretion in *Drosophila* SGs begins with fusion pore formation and redistribution of PIP_2_, followed by directional actin recruitment to secretory vesicle membranes and, finally, myosin recruitment. Moreover, secretion of the cargo is followed by complete membrane integration of secretory vesicles with the apical PM. Therefore, our real-time imaging system provides spatial and temporal resolution to image dynamic events occurring on the membranes of individual secretory granules in a living organ.

### Arp2/3 F-actin formation is required for secretion

**Figure 3:**
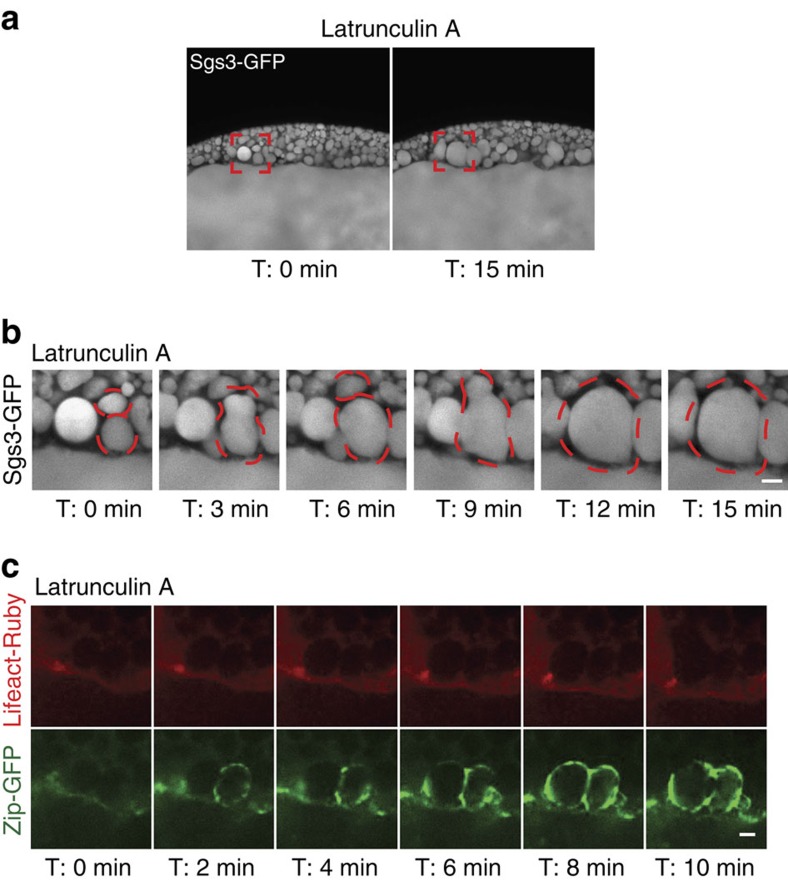
Actin is required for secretion and inhibition of compound vesicle fusion. (**a**) Secreting glands expressing Sgs3-GFP (white) were treated with latrunculin A and individual granule fusion events were imaged in real time. Red boxes denote areas shown as magnified images in **b**. (**b**) Drug treatment resulted in compound fusion events between granules that continue to expand and fail to collapse once fused with the apical membrane. Dashed red lines outline secretory granules undergoing expansion and compound fusion events. A representative times series from three independent experiments is shown. (**c**) Secreting glands expressing Lifeact-Ruby (red) and Zip-GFP (green) treated with latrunculin A show that actin is no longer recruited to granules that have fused with the apical membrane. A representative time series from four independent experiments is shown. All scale bars, 5 μm. Complete movies for each time series can be found in the Supplementary Movie section.

The structural complexities associated with large, highly glycosylated cargo such as mucins^43^,^44^, may require actomyosin contractile forces for cargo expulsion and membrane integration. We therefore examined the role of F-actin in SG secretion. We had previously shown that pharmacological disruption of the actin cytoskeleton by latrunculin A (LA) or cytochalasin D (CD) treatment impairs the gradual collapse of the secretory granules in rodent SGs *in vivo*^16^. Likewise, treatment of *Drosophila* SGs with LA or CD also resulted in disrupted secretion (Fig. 3a,b, Supplementary Fig. 5a,c and Supplementary Movies 9–12). Treatment with either drug severely inhibited vesicle collapse as fused vesicles expanded dramatically in size, underwent compound fusion and failed to undergo membrane integration (Fig. 3a,b, Supplementary Fig. 5a,c and Supplementary Movies 9–12). The efficacy of each treatment on the actin cytoskeleton was assessed by examining both Lifeact-Ruby in real time and by staining fixed glands with phalloidin (Fig. 3c, Supplementary Figs 4a,b and 5b and Supplementary Movies 13–16). Taken together, these results demonstrate a requirement for F-actin in regulated secretion in this system and highlight its role in vesicle collapse and the prevention of compound fusion.

**Figure 4:**
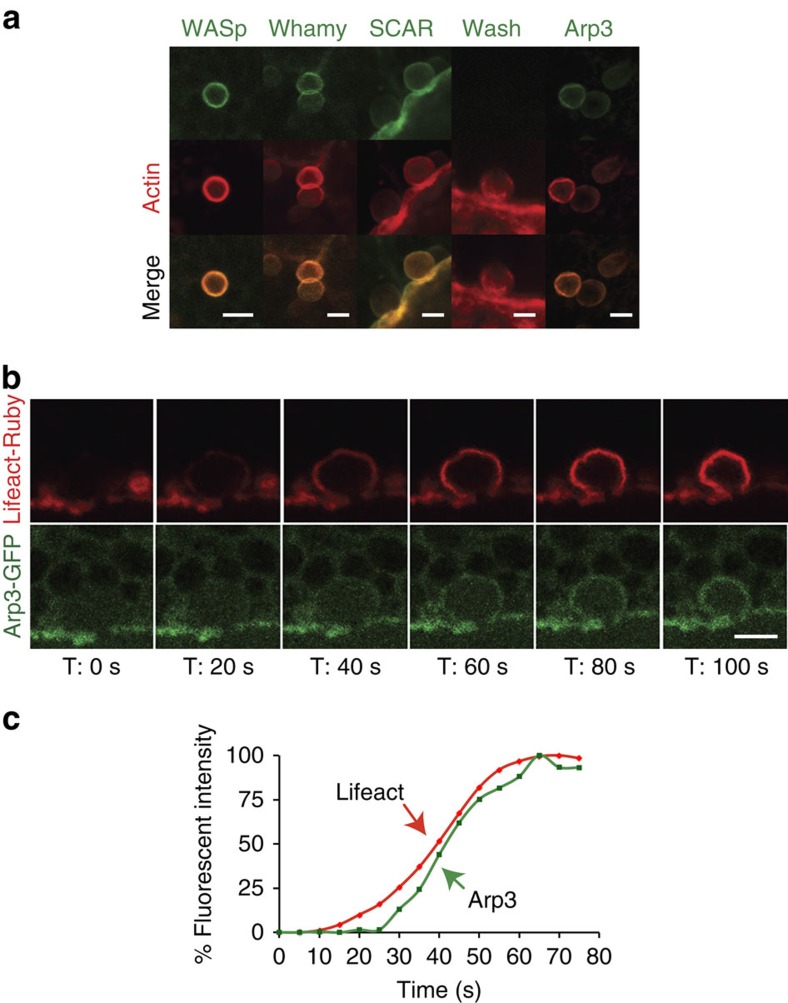
Arp2/3 and WASp are recruited to granules undergoing secretion. (**a**) Secreting glands were fixed and stained for Arp3, WASp, Whamy, SCAR or Wash (green) along with phalloidin (red). Merged images are shown in yellow. Representative images from three independent experiments are shown. (**b**) Secreting glands expressing both Arp3-GFP (green) and Lifeact-Ruby (red) were imaged in real time to show that actin is detected on fused granules before Arp3, suggesting that linear actin first coats fused granules, followed by branched actin formation. A representative time series from 11 independent experiments is shown. (**c**) Quantification of actin recruitment relative to Arp3 recruitment to fused granules. Representative graph plotting percent fluorescent intensity of each marker as a function of time for an individual secretory event is shown. Quantification was performed on 13 individual secretory events from four independent experiments. Additional graphs are shown in Supplementary Fig. 3b. All scale bars, 5 μm. Complete movies for each time series can be found in the Supplementary Movie section.

Conventional light microscopy cannot resolve the organization of actin filaments on the surface of the membranes of the secretory granules in *Drosophila* SGs. Nonetheless, based on the fact that F-actin on the granules prevents compound exocytosis, we speculated that filaments may be tightly organized around the membranes, possibly by employing extensive branching. Branched actin is nucleated from linear filaments via the Arp2/3 complex and NPFs. To determine which of these proteins may be involved in regulated secretion within *Drosophila* SGs, we performed immunofluorescent staining on actively secreting glands. Arp3, WASp, Whamy and SCAR (but not Wash) each co-localized with phalloidin on granules that had fused with the apical PM, suggesting a role for these factors in secretion (Fig. 4a). We next examined the temporal order of recruitment of the Arp3 complex onto the secreting granules by creating a *Drosophila* line expressing both GFP-labelled Arp3 (Arp3-GFP) and Lifeact-Ruby. Real-time imaging of Lifeact-Ruby and Arp3-GFP during secretion revealed that F-actin is seen on granules before Arp3 recruitment (Fig. 4b,c Supplementary Fig. 3b and Supplementary Movie 17). The average time difference in the detection of F-actin relative to Arp3 was 29±14 s (*n*=13). As the Arp2/3 complex is known to bind linear actin and is required for branched actin formation, our results suggest a model where fused vesicles are initially coated with linear actin, which is detected by Lifeact, and then subsequently bound by Arp2/3 to form a branched actin network.

**Figure 5:**
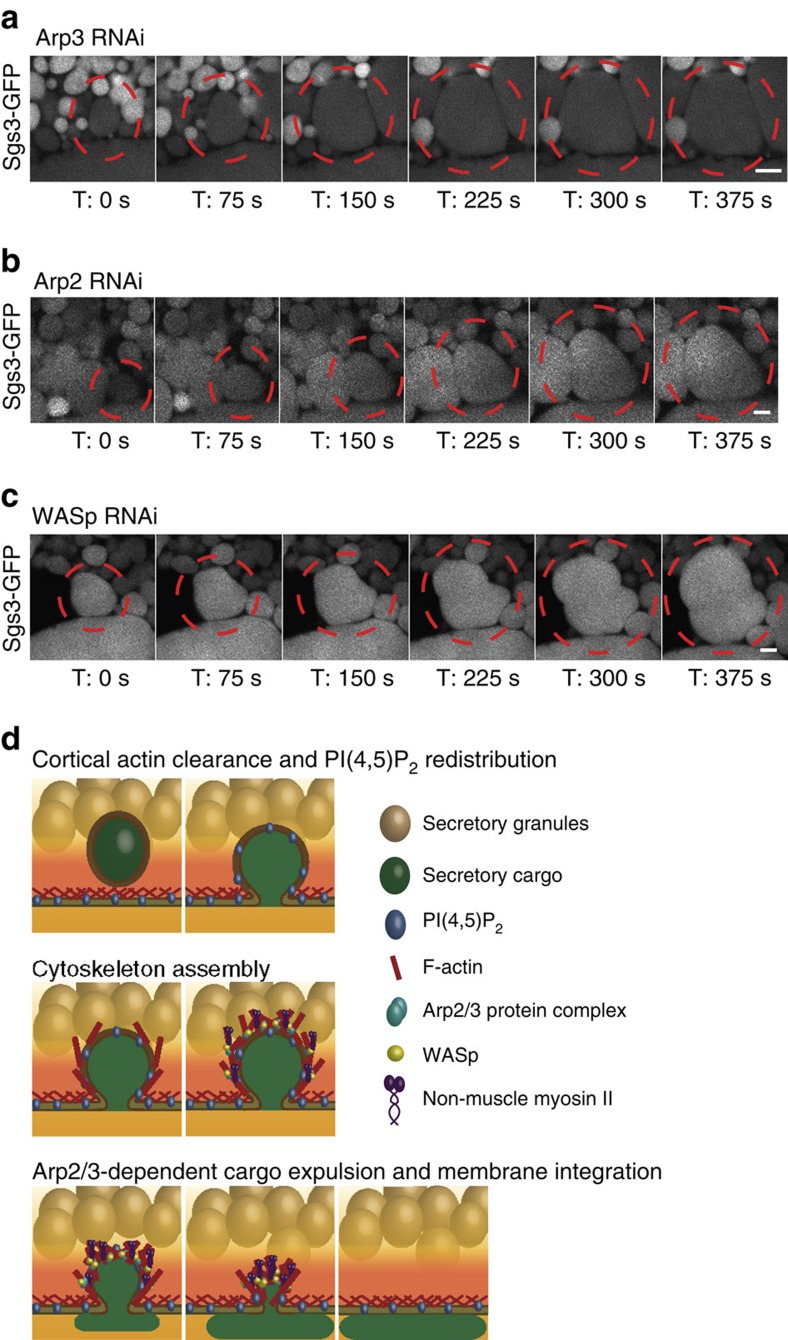
Arp2/3 and WASp are required for cargo secretion and membrane integration. (**a**) *In vivo* RNAi to *Arp3* was performed in *Drosophila* lines that also express Sgs3-GFP (white) and secretion was imaged in real time. Knockdown of *Arp3* resulted in granules that fused with the apical membrane but failed to collapse and secrete their contents. A representative time series from three independent experiments is shown. (**b**) *In vivo* RNAi to *Arp2* was performed in *Drosophila* lines that also express Sgs3-GFP (white) and secretion was imaged in real time. Loss of *Arp2* mimicked the secretion defects seen upon loss of *Arp3*. A representative time series from three independent experiments is shown. (**c**) *In vivo* RNAi to *WASp* was performed in *Drosophila* lines that also express Sgs3-GFP (white) and secretion was imaged in real time. Loss of *WASp* mimicked the secretion defects see upon loss of either *Arp2* or *Arp3*. A representative time series from four independent experiments is shown. All scale bars, 5 μm. Complete movies for each time series can be found in the Supplementary Movie section. (**d**) Model of temporal and spatial events occurring during regulated exocytosis in *Drosophila* SGs. Fully formed secretory granules reside in the secretory cells of third instar larval SGs until secretion is triggered by a pulse of 20E. The early events of secretion involve cortical actin clearance along with fusion pore formation and redistribution of apical PIP_2_ to the vesicle membranes. The exact order of these events is not known. Following these early events, components of the actin cytoskeleton are assembled on fused granules. First, linear actin is recruited followed by the recruitment of the Arp2/3 protein complex, which forms branched actin structures from existing linear filaments. Actin recruitment occurs in a directional manner originating from the PM. Actin recruitment is followed by recruitment of non-muscle myosin II. Finally, expulsion of cargo from secretory granules and integration of granular membrane with the apical PM is dependent on Arp2-, Arp3- and WASp-mediated actin formation.

To investigate whether branched actin is required for regulated exocytosis, we next performed *in vivo* knockdown of components of the Arp2/3 complex in flies expressing Sgs3-GFP or Lifeact-Ruby. RNAi to *Arp3* substantially decreased *Arp3* expression and resulted in granules that fused with the apical membrane, but failed to secrete cargo and failed to undergo membrane integration (Fig. 5a Supplementary Figs 6a,b and 7a and Supplementary Movies 18 and 19). Upon loss of *Arp3*, fused granules continued to enlarge, similar to what was observed in SGs treated with LA. However, unlike LA treatment, no evidence of compound exocytosis was seen, suggesting that linear actin still present on granules may function to block compound fusion. Interestingly, some F-actin (likely linear actin) was still detected on fused granules in the *Arp3* RNAi background, although no longer in a homogenous distribution (Supplementary Fig. 6c, d and Supplementary Movies 20 and 21). Likewise, RNAi to *Arp2* (which forms a functional complex with Arp3) phenocopied the knockdown of *Arp3*, adding further support for the role of Arp2/3-mediated branched actin in proper cargo expulsion and membrane integration (Fig. 5b and Supplementary Figs 6a, b and 7b and Supplementary Movies 22 and 23). We next examined the role of the Arp2/3 activator, WASp. RNAi to *WASp,* an NPF that can activate Arp2/3, also resulted in granules that grew larger in size, failed to expel cargo and failed to undergo membrane integration; these granules also maintained limited and unevenly distributed staining for F-actin (likely detecting linear actin) and did not show evidence of compound fusion (Fig. 5c and Supplementary Figs 6a,b,c,e and 7c and Supplementary Movies 24–27). Finally, large, expanded granules could also be seen in the SGs in the intact pupae in *Arp3* RNAi, *Arp2* RNAi or *WASp* RNAi backgrounds (Supplementary Fig. 8), again indicating that our *ex vivo* SG system faithfully recapitulates what is occurring *in vivo*. Taken together, our studies demonstrate an essential role for the branched actin nucleators Arp2, Arp3 and WASp in secretory cargo expulsion and membrane integration during regulated exocytosis *in vivo* (Fig. 5d).

## Discussion

Here, we show temporal and spatial dynamics of F-actin formation during regulated exocytosis and define essential roles for the branched actin nucleators Arp2, Arp3 and WASp in cargo secretion and membrane integration (Fig. 5d). By imaging actin dynamics in four dimensions at single granule resolution in a secreting organ, we demonstrate that actin along the PM is dynamically cleared at the point of vesicle fusion. This observation supports a role for cortical actin acting as a barrier to premature vesicle fusion, as has been proposed for other modes of secretion^4^,^5^,^6^,^45^. Shortly after this cortical rearrangement, actin begins to expand over the secretory vesicle from the site of PM contact. Previous studies imaging actin (as it is involved in exocytosis) in two dimensions suggest that recruitment occurs simultaneously over all regions of the vesicle^46^. However, our 4D renderings provide spatial and temporal resolution that has not been seen previously and show that actin recruitment to the secretory vesicle begins at the apical PM. These dynamic changes in actin organization suggest a highly ordered process whereby factors on the PM and vesicle act in concert to remodel actin to mediate secretory vesicle docking/fusion as well as to reform the actomyosin machinery around the fused vesicle. This mode of actin recruitment may provide the continuity with cortical actin required to generate the appropriate forces needed for cargo expulsion/release, especially in cases of large, highly glycosylated cargo, such as mucins.

Actin recruitment to secretory vesicles was preceded by the formation of the fusion pore between the apical PM and the secretory vesicle membrane. In addition, membrane mixing and redistribution of the membrane lipid PIP_2_ also occurred before F-actin recruitment. As PIP_2_ is known to mediate actin formation in other systems^38^,^39^, our results suggest that this membrane mixing may trigger actin polymerization on secretory granule membranes. Interestingly, actin polymerization appears to occur in two distinct steps, as the recruitment of Lifeact to secretory granules is detected before Arp3 recruitment. As the Arp2/3 complex is known to bind linear actin, this suggests that actin formation on fused secretory granules begins with linear actin formation (detected by Lifeact), which originates from the apical PM. Linear actin is then bound by the Arp2/3 complex, which catalyses the formation of branched actin. Myosin recruitment occurs after actin is present, as has been seen in cell-based systems^12^. Future work will employ this system to temporally dissect the recruitment of other factors involved in secretion.

The ordered recruitment of linear and branched forms of actin suggests essential roles for each in regulated secretion. However, specific roles for branched actin in regulated secretion were previously unknown. Taking advantage of genetic tools, we directly interrogated the role of branched actin in regulated secretion by performing RNAi to the genes that regulate its formation. *In vivo* knockdown of *Arp2* or *Arp3*, both of which are involved in the synthesis of branched actin, resulted in similar phenotypes, where secretory vesicles fused with the PM but were unable to secrete their cargo and unable to undergo membrane integration. These vesicles grew very large and in some instances, separated from the PM and floated back into the cytoplasm. In addition, *in vivo* knockdown of the NPF WASp resulted in similar phenotypes, identifying it as an additional regulator of exocytosis in this system. Taken together, our results suggest that branched actin is required to generate the force necessary to complete expulsion of the secretory cargo and integration of vesicular membranes with the PM. In the absence of branched actin, the fused secretory vesicles continue to expand in size, likely due to the hydration and expansion of the highly glycosylated mucinous cargo present within. These results have implications for the importance of branched actin formation in other systems such as the digestive tract, where secretion of large, highly glycosylated/cross-linked cargo is essential for extracellular matrix formation, microbial interactions and protection of epithelial cell surfaces^47^,^48^.

Our results also suggest unique roles for linear actin in regulated exocytosis. LA and CD, which disrupt the formation of both linear and branched actin, resulted in compound fusion of secretory vesicles with one another, which was not seen upon knockdown of *Arp2, Arp3* or *WASp*. This suggests that the linear actin first recruited to the fused secretory vesicle may act as a barrier to prevent the fusion of other secretory vesicles. However, we cannot rule out the possibility that residual branched actin present in our knockdown animals may be able to prevent compound fusion events. Future studies combining fluorescent imaging and electron microscopy of genetically manipulated SGs will aim to identify specific actin structures present on fused secretory granules and how they influence vesicle interactions.

Our study is unique in that we image secretory cargo as a direct indicator of secretion. Previous studies have used visualization of the fusion pore as evidence of cargo secretion^8^. However, in our system, fusion pore formation and cargo expulsion are temporally distinct events, indicating that fusion pore formation alone is not sufficient for secretion of vesicle contents. In addition to the forces mediated by branched actin, other factors may be required for the secretion of large, glycosylated cargos. This system can be used to interrogate other genes that regulate the processing, packaging and release of complex cargos.

In summary, our study highlights coordinated actin clearance and directional recruitment from the PM as well as essential roles for branched actin nucleators in regulated exocytosis in a living, secreting organ. The powerful combination of *Drosophila* genetics with *in vivo* and *ex vivo* imaging allows one to rapidly interrogate the role of factors in many aspects of secretion, including vesicle biogenesis, vesicle movement, fusion with the PM, release of granule cargo and expansion of cargo once in the lumen. Given that many genes and mechanisms involved in secretion are conserved across species, this system can provide insight into the factors that regulate mammalian secretion.

## Methods

### Fly strains and genetics

The following *Drosophila* lines were used: Bloomington #5884 (w*; P{Sgs3-GFP}2)^32^, #5885 (w*; P{Sgs3-GFP}3), #35544 (y^1^ w*; P{UAS-Lifeact-GFP}VIE-260B), #35545 (y^1^ w*; P{UAS-Lifeact-Ruby}VIE-19A), #7118 (*w^1118^; P{UAS-myr-mRFP}1*), #32221 (w*; P{10XUAS-IVS-myr::tdTomato}attP2), #39723 (w*; P{UASp-Arp3.GFP}2)^49^, #39693 (y^1^ w*; P{UAS-PLCδ-PH-EGFP}3), #58362 (w*; P{UASt-Lifeact-RFP}3). RNAi lines containing upstream activating sequence (UAS)-inducible inverted repeats (IR) were from the Vienna Drosophila RNAi Center (VDRC)^50^ and were the following stocks: VDRC #29944 (*P{UAS-Arp2IR*), VDRC #108951 (*P{UAS-Arp3IR*), VDRC #108220 (*P{UAS-WaspIR*), VDRC #60008 (*w^1118^; P{UAS-dicer2^w+^}*) and VDRC 60009 (*w^1118^; P{UAS-dicer2^w+^}*). The wild-type stocks used were either *Oregon R* or *w^1118^* (VDRC #60000). The Gal4 driver line used in these studies was Bloomington stock #6978 (*w^1118^; P{GawB}c135*), which drives expression in the *Drosophila* SG beginning during embryonic stage 15 and continuing through the third instar larval stage. The *c135-Gal4* driver stock was recombined with *Sgs3-GFP* to generate *c135-Gal4>Sgs3-GFP*. VDRC #60008, which expresses *dicer2* under the control of the UAS promoter, was crossed to VDRC #108951 and VDRC #108220 to enhance RNAi-mediated knockdown. VDRC #60009, which also expresses *dicer2*, was crossed to VDRC #29944 to enhance gene knockdown. *Sgs3-GFP* was recombined with Bloomington #3554 to generate *Sgs3-GFP, UAS-Lifeact-Ruby. Sgs3-GFP* was recombined with Bloomington #3222 1to generate *Sgs3-GFP*, *UAS-Myr-tdTomato*. Bloomington #3554 was recombined with Flytrap line #CC01626 to generate *Zip-GFP*, *UAS-Lifeact-Ruby*. The *c135-Gal4* driver was recombined with *Zip-GFP, UAS-Lifeact-Ruby* to generate *c135-Gal4>Zip-GFP, UAS-Lifeact-Ruby*. Bloomington #3554 was recombined with Bloomington #39723 to generate *UAS-Arp3-GFP, UAS-Lifeact-Ruby*. The *c135-Gal4* driver was recombined with Bloomington #58362 to generate *c135-Gal4> UAS-Lifeact-RFP*. All *Drosophila* crosses were kept on MM media (KD Medical, Inc.) at 25 °C unless specified otherwise. Crosses to generate expression of dsRNA were performed using flies from a *UAS-IR* transgenic line and the *c135-Gal4>Sgs3-GFP* or *c135-Gal4>Zip-GFP, UAS-Lifeact-Ruby* stocks.

### *Ex vivo* SG culture and real-time imaging

SGs from third instar wandering larvae were dissected in Schneider's *Drosophila* medium (Gibco) and transferred to glass bottom culture dishes (MatTek) containing 50 μl media. To immediately image glands that were actively secreting, the majority of the media were then removed from the dish and a 13-mm, 0.05 μm, polycarbonate membrane filter (Sterlitech) was gently placed on top of the gland. The media that were removed from the dish were then placed on top of the membrane filter and the sample was subsequently imaged. For imaging sessions longer than 2 h, 0.5% low melt agarose in Schneider's *Drosophila* medium was layered on top of glands and the sample was subsequently imaged. To induce secretion in non-secreting glands, 20-hydroxyecdysone (Sigma) was added to glands in glass bottom dishes to a final concentration of 700 μM and dishes were placed in a humidified chamber at room temperature for 90 min. The media from these dishes were then removed, a membrane was gently placed on top of the glands and media were added back to the top of the membrane. For experiments involving fluorescent dextran, SGs from non-secreting third instar wandering larvae were dissected and transferred to glass bottom culture dishes containing 50 μl media. Alexa Fluor 647, Alexa Fluor 680 or Rhodamine B Dextran (Invitrogen) was subsequently added to the media to a final concentration of 1 mM and then nutated at room temperature for 45 min. 20-Hydroxyecdysone was then added to a final concentration of 700 μM and placed in a humidified chamber at room temperature for 90 min. The media from these dishes were then removed, a membrane was gently placed on top of the glands and media were added back to the top of the membrane. For inhibitor experiments, LA (final concentration 3 μM) or CD (final concentration 10 μM) was added to the media resting on top of the polycarbonate membrane immediately before imaging. All glands were imaged on either a Zeiss LSM 510, Zeiss LSM 700 with a C-Apochromat × 63/1.2 W Corr UV-VIS-IR objective or a Nikon A1R+ confocal microscope with both CFI Lambda S Apo LWD × 40/1.15 numerical aperture (NA) water immersion (WI) and CFI Plan Apo IR × 60/1.27 NA WI objectives.

### *In vivo* SG imaging

Wandering third instar larvae were immobilized between two 24 × 50 mm^2^ coverslips in sufficient Schneider's media such that the liquid spread evenly across the entire area of the coverslip. Imaging was performed on a Nikon A1R+ confocal microscope, with × 10 Plan Apo 0.45 NA or × 20 Plan Apo VC 0.75 NA objective, using the ‘keep object in view' function of the advanced tracking module to compensate for *X*–*Y* movement. Single confocal sections were acquired every 5 s to keep glands in the imaging window and a Z-stack was imaged every minute to accommodate movement in the *Z* plane.

### Imaging of prepupae

Prepupae were initially photographed in original MM media vials used to set up crosses. Images were acquired on a Leica MZ 16F fluorescent stereomicroscope with DC 500 camera. Following low-magnification imaging, the prepupae were then transferred to glass bottom culture dishes (MatTek) containing 50 μl media. A 13-mm, 0.05 μm, polycarbonate membrane filter (Sterlitech) was gently placed on top of the prepupae and used to roll the sample such that SGs were closest to the cover glass. Z-stacks were acquired on a Nikon A1R+ confocal microscope with a × 20 Plan Apo VC 0.75 NA objective.

### SG fixation and staining

SGs from third instar wandering larvae were dissected in Schneider's *Drosophila* Medium (Gibco) and immediately transferred to 4% paraformaldehyde in PBS. Glands were fixed for 20 min at 4 °C. Samples were then washed three times in PBST (PBS, 0.1% Triton X-100) and transferred to blocking buffer (2% Goat serum/PBS, 0.1% Triton X-100) for 1 h at room temperature on a rocker. Samples were then incubated with primary antibodies overnight at 4 °C. Primary antibodies used were mouse anti-WASp (P5E1-Developmental Studies Hybridoma Bank, 1:10), mouse anti-Wash (P3H3-Developmental Studies Hybridoma Bank, 1:20), mouse anti-SCAR (P1C1-Developmental Studies Hybridoma Bank, 1:50), mouse anti-Whamy (P4A8-Developmental Studies Hybridoma Bank, 1:20)^23^ and rabbit anti-Arp3 (the kind gift of Dr W. Theurkauf, 1:500) (ref. 51). Samples were then washed three times for 15 min each in PBST and incubated with secondary antibodies for 3 h at room temperature on a rocker. Alexa Fluor 488- or Alexa Fluor 647-conjugated secondary antibodies (Invitrogen, 1:50) and TRITC-conjugated phalloidin (Sigma, 1:100) were used. Samples were then washed three times for 15 min each in PBST, mounted in aqueous mounting medium (Electron Microscopy Sciences) and imaged using a Zeiss LSM 700 confocal microscope.

### Quantitative RT–PCR

Quantitative RT–PCR was used to determine efficiency of gene silencing when RNAi was induced. cDNA prepared from SGs from third instar wandering larvae of *c135-Gal4,Sgs3-GFP>IR* crosses or controls (*c135-Gal4,Sgs3-GFP >VDRC#60000* or *OregonR*) was used in qPCR reactions. Briefly, RNA was isolated using the RNAqueous-Micro Total RNA Isolation Kit (Ambion). cDNA synthesis was performed using iScript cDNA Synthesis Kit (Bio-Rad). PCR primers (Supplementary Fig. 7d) were designed using Beacon Designer software (Bio-Rad). QRT-PCR was performed on a CFX96 real-time PCR thermocycler (Bio-Rad) using the SYBR-Green PCR Master Mix (Bio-Rad). RNA levels were normalized to 18S rRNA. Values represent mean value±s.e.m. Significance values were calculated using two-tailed Student's *t*-test.

### Statistical analyses

Number of replicates used for each analysis is specified in figure legends. To measure percent fluorescent intensity for Lifeact/Dextran, Lifeact/PLCδ-PH, Lifeact/Zip or Lifeact/Arp3 experiments, Nikon Elements software was used. A rectangular or oval region of interest (ROI) was drawn around individual secreting granules and the Time Measurement analysis tool was used to measure average fluorescent intensity within the ROI for each channel. Values were exported to Microsoft Excel and percent fluorescent intensity was calculated. The percent fluorescent intensity of each channel at each time point was calculated as: % fluorescent intensity=(Fluorescent intensity—Fluorescent intensity of T_0_)/(Fluorescent intensity of *T*_max_—Fluorescent intensity of *T*_0_ ) × 100. *T*_0_ is the time frame at which one of the two channels being measured begins to continually increase in fluorescent intensity. *T*_max_ is the maximum fluorescent intensity value reached for each respective channel being measured. Each graph was plotted from *T*_0_ until at least two time point beyond *T*_max_ for each channel. Percent fluorescent intensity values that were negative due to fluctuations in intensity values as a result of granule movement were set to 0. Quantification was only performed on granules that did not move within or through the imaging plane during the entire process of secretion. To determine the average time difference in the detection of each component in the pairwise comparisons, we calculated the time between *T*_0_ and when fluorescent intensity of the second component began to continually increase and was at least 1% higher than the previous time point. Measurements were performed on 11 granules for Lifeact/Dextran, 12 granules for Lifeact/PLCδ-PH, 11 granules for Lifeact/Zip and 13 granules for Lifeact/Arp3. Averages were calculated for each pair and are expressed as mean values±s.d. To measure fold change in size of secreting Sgs3-GFP granules, Fiji (http://fiji.sc/Fiji) was used to outline and measure the area of individual secreting granules as a function of time after fusion. Granule fusion with the apical membrane correlates with a change in fluorescent intensity of the fusing granule. Time 0 s represents the frame immediately before a change in fluorescent intensity of a given granule is detected. Values were exported to Microsoft Excel and fold change in granule size was calculated. Graphs represent mean values of all granules measured±s.d. Quantification was only performed on granules where fusion with the apical membrane could be detected and could be clearly visualized throughout the time frame of analysis. No statistical method was used to predetermine sample size.

### Image analysis

Microscopy images were opened in Fiji and individual frames were exported as still images or Movies. Movies were subsequently formatted using Apple Final Cut Pro, Adobe Media Encoder or Apple Quicktime software. Imaris Bitplane software was used to perform surface rendering of 4D Lifeact experiments. Adobe Illustrator was used to generate figures from individual still images.

## Additional information

**How to cite this article:** Tran, D. T. *et al.* Arp2/3-mediated F-actin formation controls regulated exocytosis *in vivo*. *Nat. Commun.* 6:10098 doi: 10.1038/ncomms10098 (2015).

## Supplementary Material

Supplementary Figures Supplementary Figures 1-8

Supplementary Movie 1 Real time imaging of Drosophila secretion. Third instar larval SGs expressing Sgs3-GFP (green) were cultured ex vivo and secretion was induced by the addition of 20E. Secretory cargo is released into the apical lumen resulting in lumenal expansion over time. Scale bar=50μm.

Supplementary Movie 2 Real time imaging of apical membrane during secretion. Secreting third instar larval SGs expressing Sgs3-GFP (green) and Myr-tdTomato (red) were imaged in real time. Deformation of apical membrane can be detected at the site of granule fusion. Scale bar=5μm.

Supplementary Movie 3 Real time imaging of actin recruitment to secreting granules. Secreting third instar larval SGs expressing Sgs3-GFP (green) and Lifeact-Ruby (red) were imaged in real time. Actin is recruited to secreting granules prior to cargo expulsion. Scale bar=5μm.

Supplementary Movie 4 4D imaging of actin dynamics during secretion. Secreting third instar larval SGs expressing Lifeact-GFP (white) were imaged in 3 dimensions (3D) over time. Maximum projection view of the 3D volume shows dynamic apical actin that is cleared at the site of vesicle fusion. Subsequent actin recruitment to vesicles originates at the PM. Scale bar=5μm.

Supplementary Movie 5 Surface reconstruction of actin dynamics during secretion. Surface reconstruction of actin (white) in the immediate vicinity of an individual secretory granule during secretion. Cortical actin is cleared at the site of vesicle fusion and actin is recruited to the vesicle directionally, from the PM. Scale bar=3μm.

Supplementary Movie 6 Fusion pore forms prior to actin recruitment. Lumens of SGs expressing Lifeact-GFP (green) were infused with a fluorescent dextran (cyan) to image the moment of fusion pore formation between the apical membrane and secretory granule. Real time imaging demonstrates fusion pore formation prior to actin recruitment to the granule. Scale bar=5μm.

Supplementary Movie 7 PI(4, 5)P2 is detected on fused granules prior to actin recruitment. Secreting SGs expressing PLCδPH-EGFP (green) and Lifeact-RFP (red) were imaged in real time. PLCδPH is detected on secreting granules prior to Lifeact, demonstrating that membrane mixing and thus diffusion of PI(4, 5)P2 from the apical to fused vesicle membrane occurs prior to actin recruitment. Scale bar=5μm.

Supplementary Movie 8 Actin is recruited prior to non-muscle myosin II. Secreting SGs expressing Lifeact-Ruby (red) and Zip-GFP (green) (non-muscle myosin II) were imaged in real time, demonstrating that actin is recruited to the granule prior to myosin. Scale bar=5μm.

Supplementary Movie 9 Latrunculin A treatment blocks secretion. Secreting SGs expressing Sgs3-GFP (white) were treated with 3μM Latrunculin A to block actin polymerization. Treatment results in compound fusion events and disruption of secretion. Scale bar=5μm.

Supplementary Movie 10 Latrunculin A treatment blocks secretion. Full imaging frame view of Supplementary Movie 9. Scale bar=5μm.

Supplementary Movie 11 Cytochalasin D treatment blocks secretion. Secreting SGs expressing Sgs3-GFP (white) were treated with 10μM Cytochalasin D to block actin polymerization. Similar to Latrunculin A treatment, this results in compound fusion events and disrupted secretion. Scale bar=5μm.

Supplementary Movie 12 Cytochalasin D treatment blocks secretion. Full imaging frame view of Supplementary Movie 11. Scale bar=5μm.

Supplementary Movie 13 Latrunculin A treatment blocks actin recruitment. Secreting SGs expressing Zip-GFP (green) and Lifeact-Ruby (red) were treated with 3μM Latrunculin A to block actin polymerization. Treatment blocks actin recruitment to granules. Scale bar=5μm.

Supplementary Movie 14 Latrunculin A treatment blocks actin recruitment. Full imaging frame view of Supplementary Movie 13. Scale bar=5μm.

Supplementary Movie 15 Cytochalasin D treatment blocks actin recruitment. Secreting SGs expressing Zip-GFP (green) and Lifeact-Ruby (red) were treated with 10μM Cytochalasin D to block actin polymerization. Similar to Latrunculin A, this treatment blocks actin recruitment to granules. Scale bar=5μm.

Supplementary Movie 16 Cytochalasin D treatment blocks actin recruitment. Full imaging frame view of Supplementary Movie 15. Scale bar=5μm.

Supplementary Movie 17 Actin is recruited prior to Arp3. Secreting SGs expressing Lifeact-Ruby (red) and Arp3-GFP (green) were imaged in real time. Actin is detected on secreting granules prior to Arp3. Scale bar=5μm.

Supplementary Movie 18 Arp3 is required for cargo expulsion and membrane integration. In vivo RNAi to *Arp3* was performed in *Drosophila* lines expressing Sgs3-GFP (white). SG secretion was imaged in real time. *Arp3* knockdown resulted in granules that fused with the apical membrane but failed to secrete cargo and undergo membrane integration. Scale bar=5μm.

Supplementary Movie 19 Arp3 is required for cargo expulsion and membrane integration. Full imaging frame view of Supplementary Movie 18. Scale bar=5μm.

Supplementary Movie 20 Actin recruitment following Arp3 RNAi. In vivo RNAi to *Arp3* was performed *Drosophila* lines expressing Lifeact-Ruby (red). SG secretion was imaged in real time, demonstrating irregular recruitment of actin. Scale bar=5μm.

Supplementary Movie 21 Actin recruitment following Arp3 RNAi. Full imaging frame view of Supplementary Movie 20. Scale bar=5μm.

Supplementary Movie 22 Arp2 is required for cargo expulsion and membrane integration. In vivo RNAi to *Arp2* was performed in *Drosophila* lines expressing Sgs3-GFP (white). SG secretion was imaged in real time. *Arp2* knockdown resulted in granules that fused with the apical membrane but failed to collapse. Scale bar=5μm.

Supplementary Movie 23 Arp2 is required for cargo expulsion and membrane integration. Full imaging frame view of Supplementary Movie 22. Scale bar=5μm.

Supplementary Movie 24 WASp is required for cargo expulsion and membrane integration. In vivo RNAi to *WASp* was performed in *Drosophila* lines expressing Sgs3-GFP (white). SG secretion was imaged in real time. *WASp* knockdown resulted in granules that fused with the apical membrane but failed to collapse. Scale bar=5μm.

Supplementary Movie 25 WASp is required for cargo expulsion and membrane integration. Full imaging frame view of Supplementary Movie 24. Scale bar=5μm.

Supplementary Movie 26 Actin recruitment following WASp RNAi. In vivo RNAi to *WASp* was performed in *Drosophila* lines expressing Lifeact-Ruby (red). SG secretion was imaged in real time, demonstrating irregular recruitment of actin. Scale bar=5μm.

Supplementary Movie 27 Actin recruitment following WASp RNAi. Full imaging frame view of Supplementary Movie 26. Scale bar=5μm.
