# Supplementary material for: Arp2/3-mediated F-actin formation controls regulated exocytosis in vivo
Source: Nat Commun. 2015 Dec 7;6:10098. doi: 10.1038/ncomms10098 (PMC4686765; doi:10.1038/ncomms10098)
Supplement: Supplementary Figures — 1-8 [file ncomms10098-s1.pdf]

Supplementary Figure 1

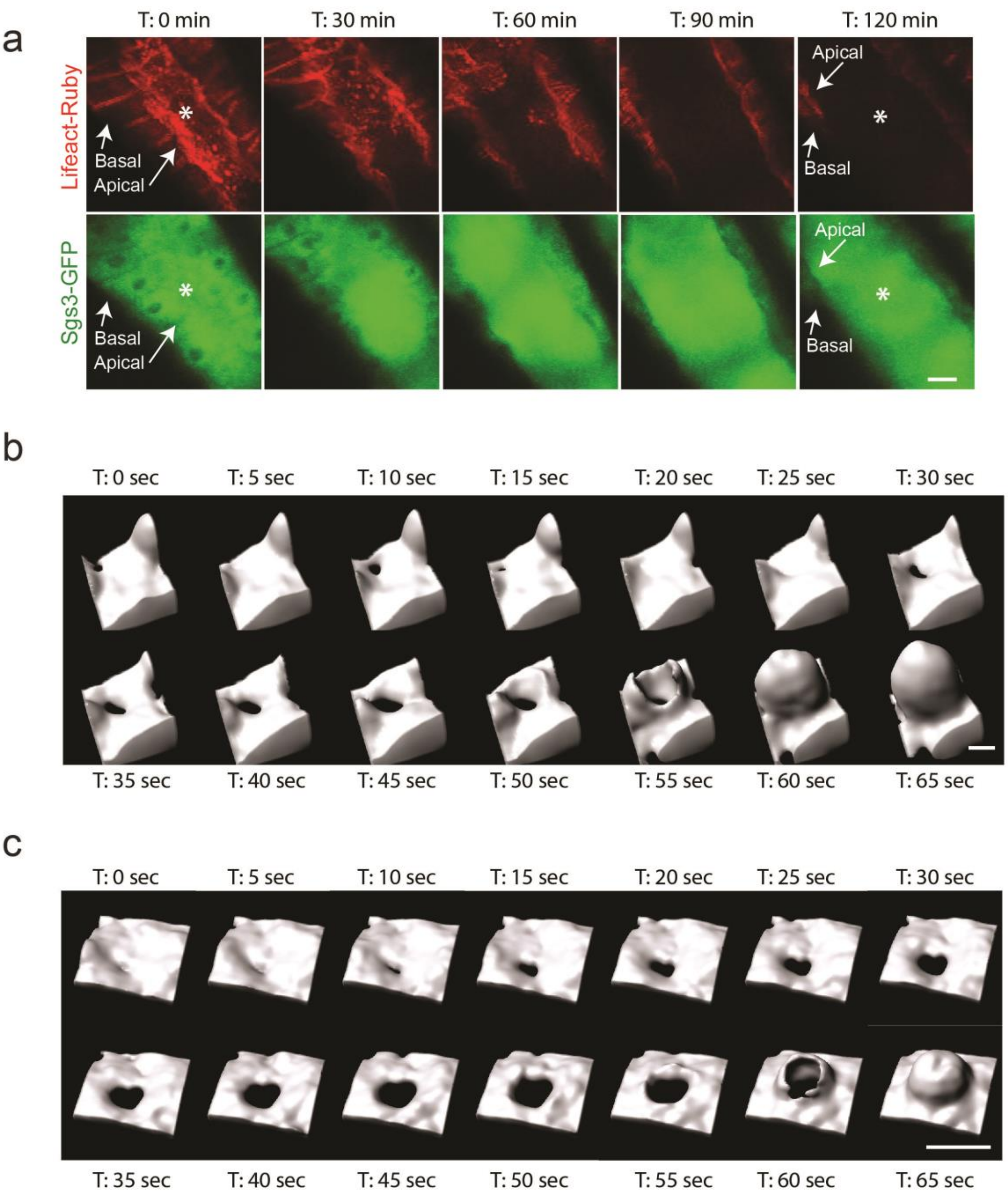

Supplementary Figure 1. Real time in vivo imaging of SG secretion. (**a**) SGs from *Drosophila* third instar larvae that express Sgs3-GFP (green) and Lifeact-Ruby (red) were imaged in vivo to visualize secretion in real time. Sgs3-GFP is secreted into the lumen over time, leading to lumenal expansion. Expansion of the apical lumen can be clearly detected by visualizing Lifeact signal. The distal tip of the gland is at the bottom right of the image. Apical and basal surfaces are labeled. The SG lumen is denoted by a *. A representative time series from 3 independent experiments is shown. Scale bar=50µm. (**b**) and (**c**) Additional surface reconstructions of 4D Lifeact-GFP (white) imaging during granule secretion showing actin clearance at the apical PM followed by actin recruitment from the PM to secretory vesicles. Representative time series from 9 granule reconstructions over 3 independent experiments are shown. Scale bar=5µm.

Supplementary Figure 2

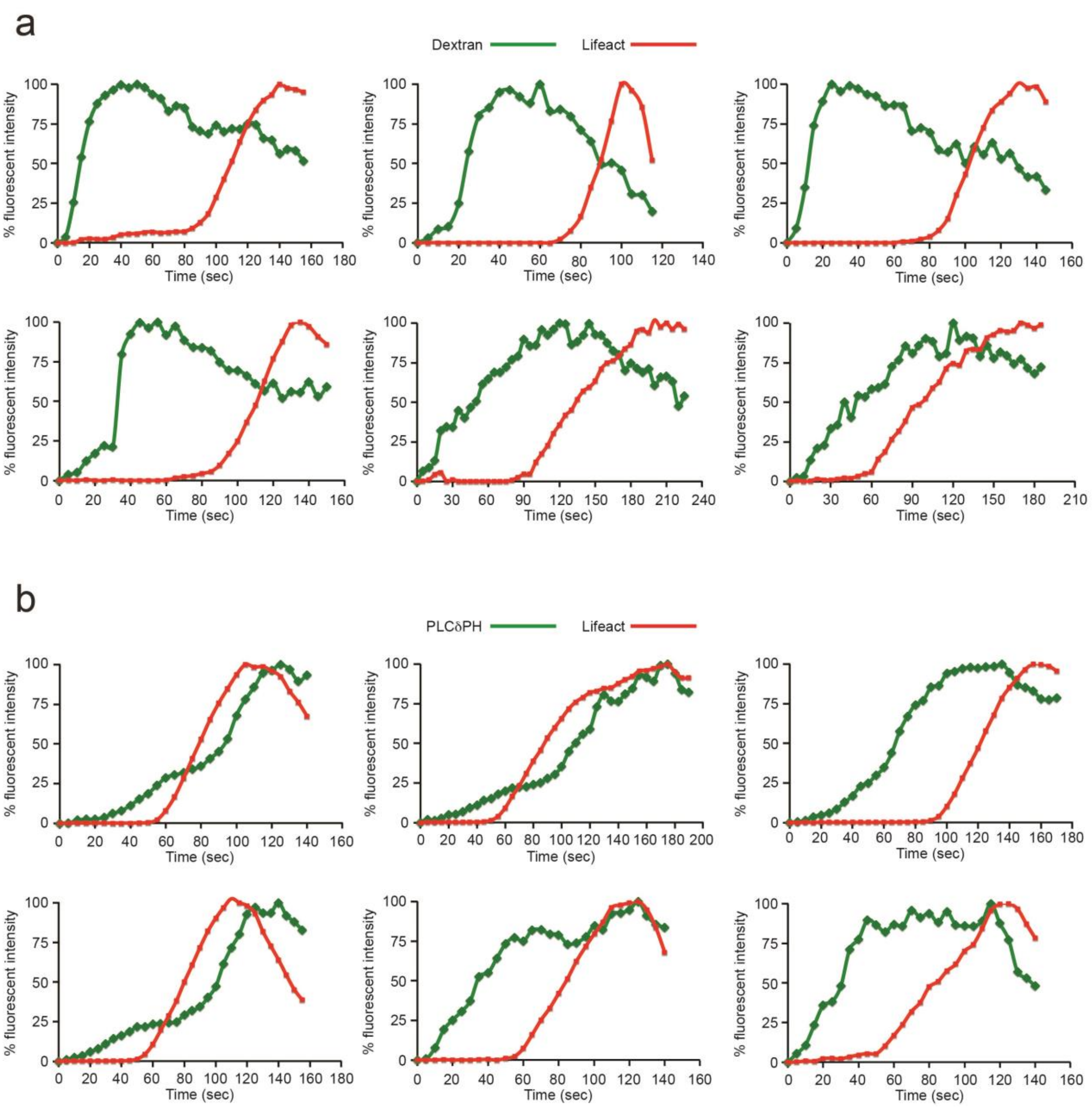

Supplementary Figure 2. Quantitation of actin recruitment to secretory vesicles relative to fusion pore formation and PI(4, 5)P_2 redistribution.

(**a**) Additional quantitation of Lifeact recruitment relative to time of dextran entry into fused granules (fusion pore formation). (**b**) Additional quantitation of PLCδPH appearance relative to Lifeact recruitment to fused granules. Representative graphs plotting percent fluorescent intensity of each marker as a function of time for individual secretory events are shown. Quantitation was performed on 11 individual secretory events over 2 independent experiments and 12 individual secretory events over 2 independent experiments, respectively.

Supplementary Figure 3

a

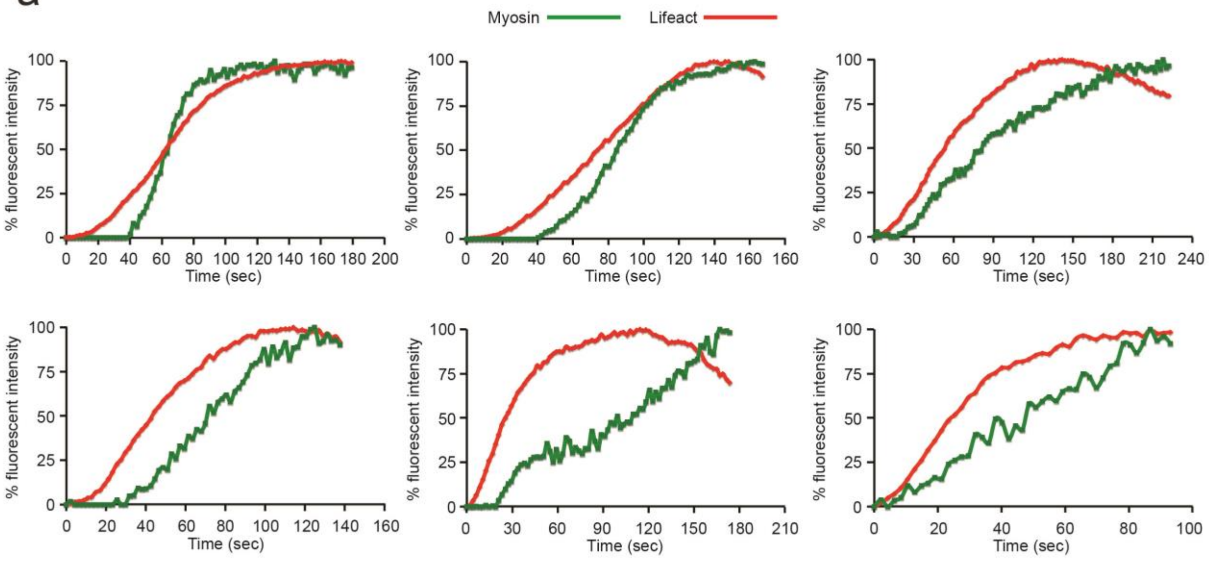

b

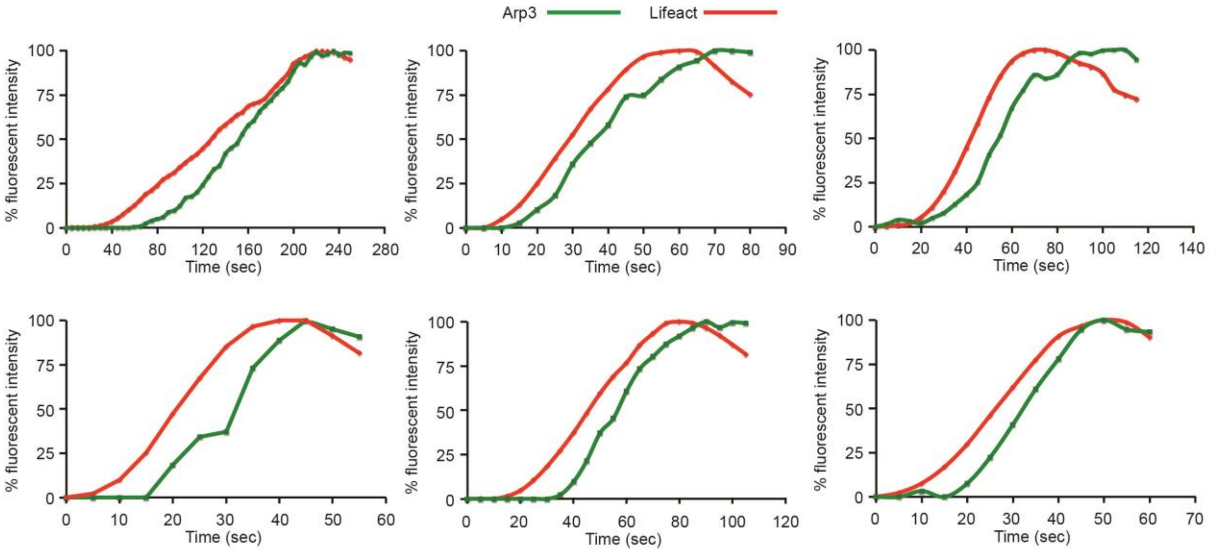

Supplementary Figure 3. Quantitation of actin recruitment to secretory vesicles relative to myosin recruitment and Arp3 recruitment.

(**a**) Additional quantitation of Lifeact recruitment relative to myosin recruitment to fused granules. (**b**) Additional quantitation of Lifeact recruitment relative to Arp3 recruitment to fused granules. Representative graphs plotting percent fluorescent intensity of each marker as a function of time for individual secretory events are shown. Quantitation was performed on 11 individual secretory events over 4 independent experiments and 13 individual secretory events from 4 independent experiments, respectively.

Supplementary Figure 4

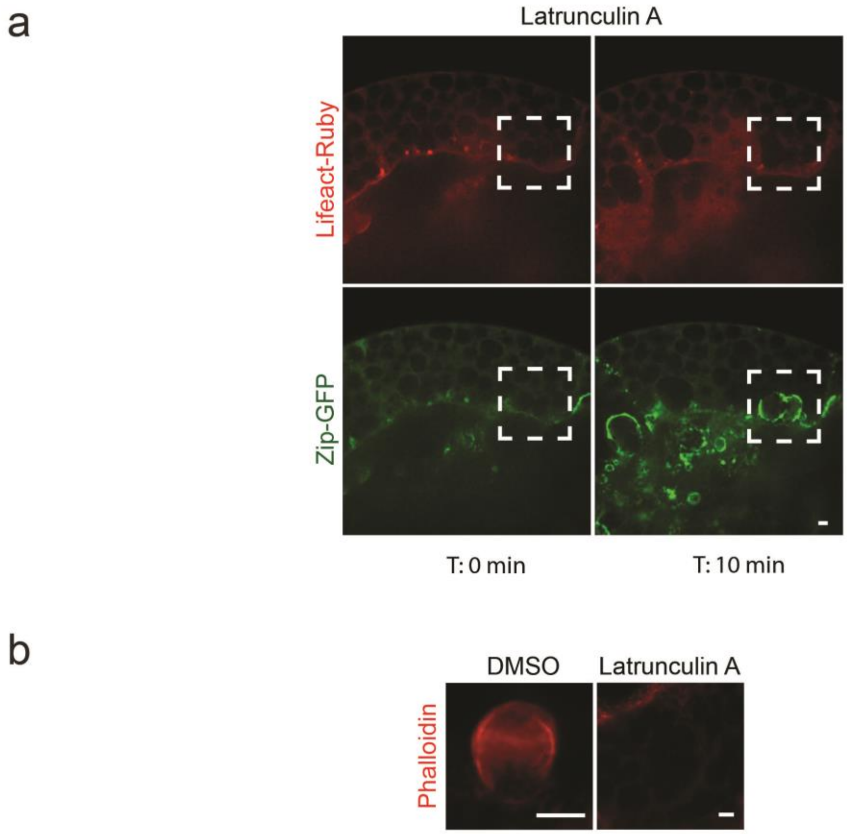

Supplementary Figure 4. Latrunculin A treatment blocks actin recruitment. (**a**) Full imaging frame view of glands expressing Lifeact-Ruby (red) and Zip-GFP (green) treated with latrunculin A. White boxes denote areas shown as magnified images in Fig. 3c. A representative time series from 3 independent experiments is shown. (**b**) Secreting SGs were treated with either DMSO or Latrunculin A for 30 min, then fixed and stained with TRITC-phalloidin (red). Representative images from 3 independent experiments are shown. All scale bars=5μm.

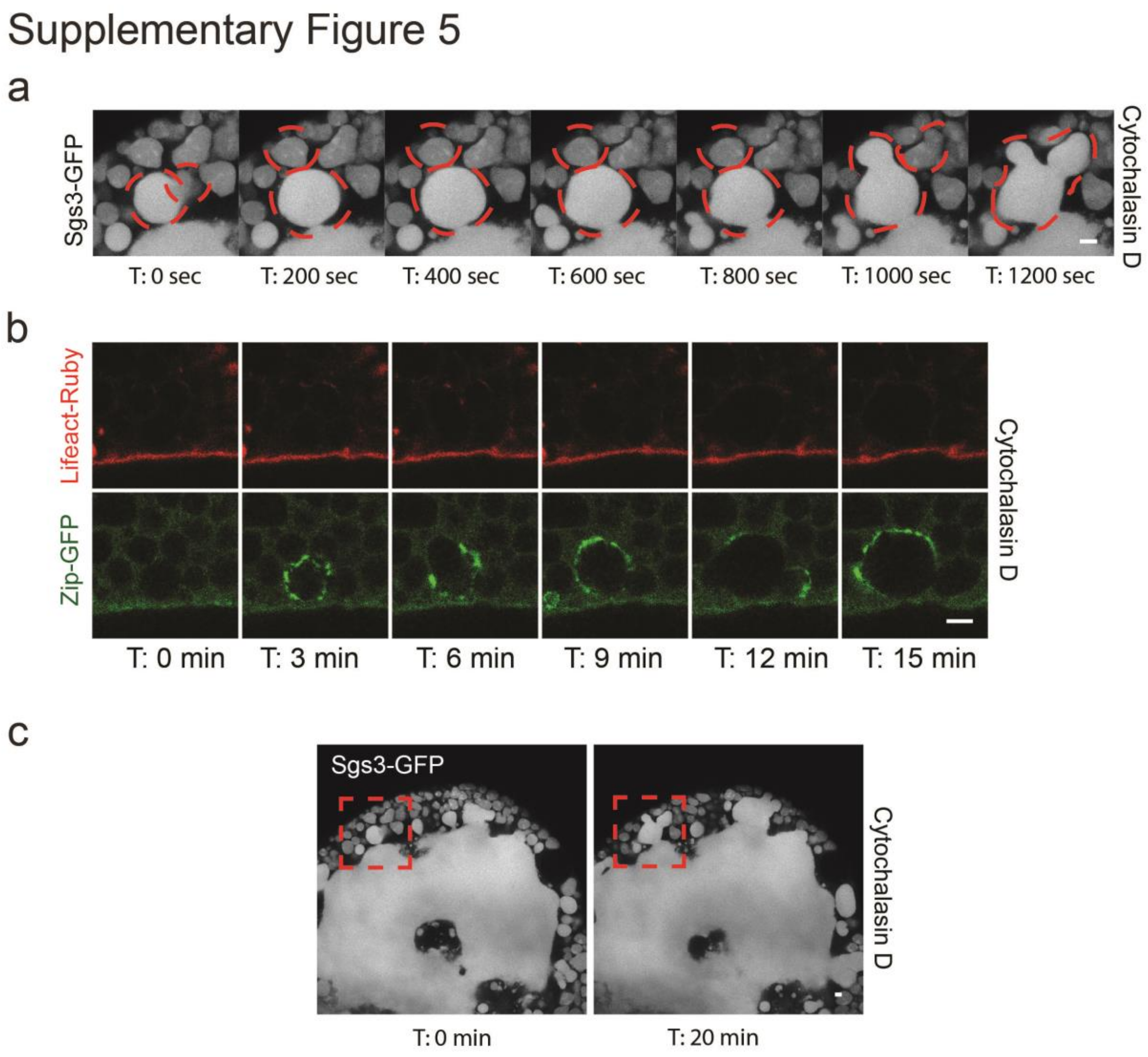

Supplementary Figure 5. Cytochalasin D treatment blocks SG secretion. (**a**) Secreting glands expressing Sgs3-GFP (white) were treated with cytochalasin D and individual fusion events were imaged in real time. Dashed red lines outline secretory granules undergoing expansion and compound fusion events. A representative time series from 3 independent experiments is shown. (**b**) Secreting glands expressing Lifeact-Ruby (red) and Zip-GFP (green) treated with cytochalasin D demonstrate that actin is not recruited to granules that have fused with the apical membrane. A representative time series from 3 independent experiments is shown. (**c**) Full imaging frame view of experiment shown in (**a**). Red boxes represent magnified area shown in (**a**). All scale bars=5µm.

Supplementary Figure 6

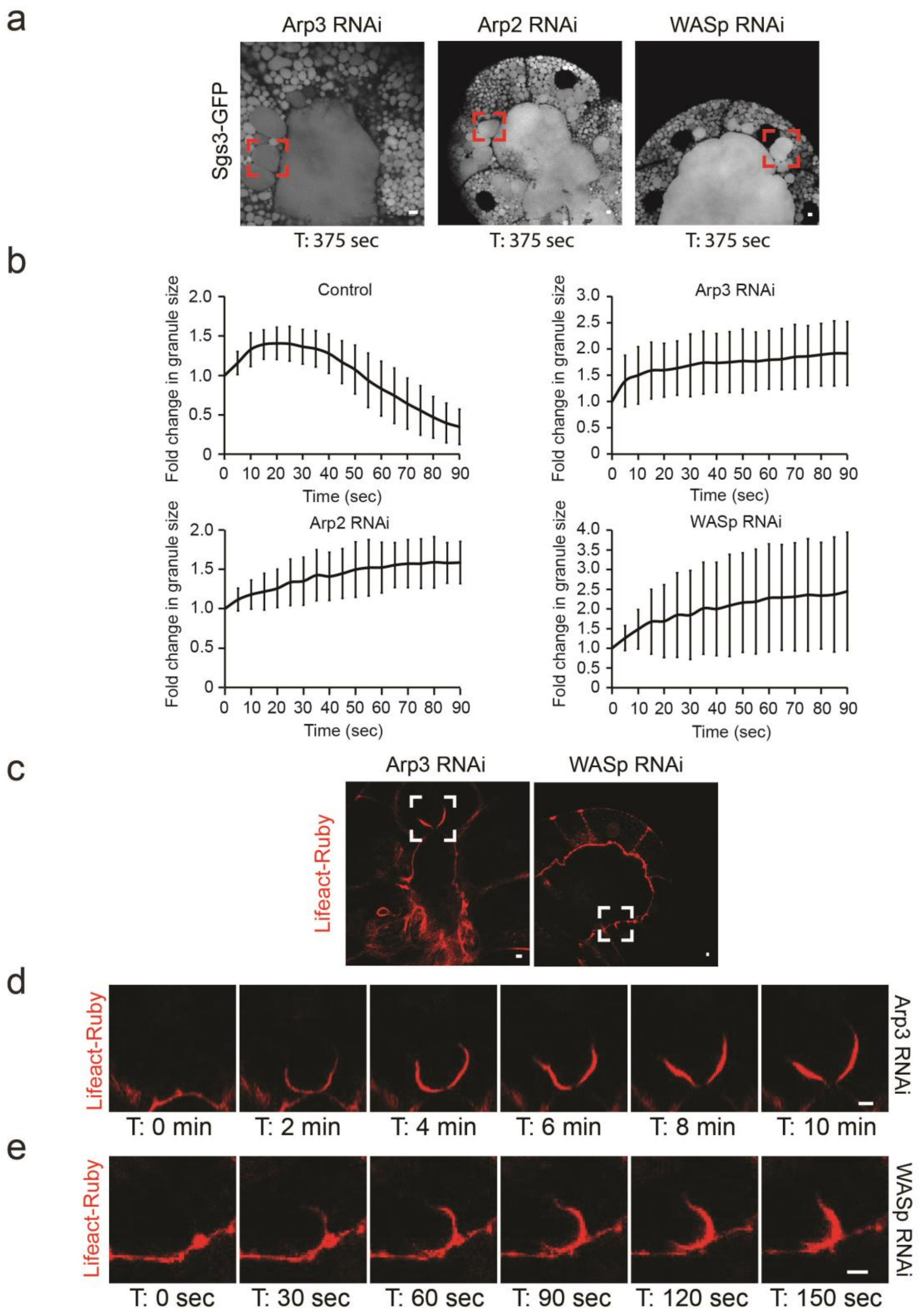

Supplementary Figure 6. Arp2/3 and WASp are required for secretion and membrane integration. (**a**) In vivo RNAi to *Arp3, Arp2* or *WASp* was performed in *Drosophila* lines that also express Sgs3-GFP (white) and secretion in third instar larval SGs was imaged in real time. Full imaging frame view of glands is shown, where red boxes highlight magnified regions shown in Fig. 5 a-c. Representative images from 3 (*Arp3* RNAi), 3 (*Arp2* RNAi), and 4 (*WASp* RNAi) independent experiments are shown. (**b**) Quantitation of fold change in granule size as function of time after fusion with the PM was performed and graphed. Granule fusion with the PM corresponds to a change in fluorescent intensity of Sgs3-

GFP in the fusing granule. Time 0 s represents the frame immediately before a detectable change in intensity. The following number of replicates were performed: for control glands, 15 individual secretory events from 4 independent experiments; for *Arp3* RNAi glands, 14 individual secretory events from 3 independent experiments; for *Arp2* RNAi glands, 13 granules from 3 independent experiments; for *WASp* RNAi glands, 12 individual secretory events from 3 independent experiments. Mean values are shown ± s.d. (**c**) In vivo RNAi to *Arp3* or *WASp* was performed in *Drosophila* lines that also express Lifeact-Ruby (red) to image actin dynamics. White boxes represent magnified regions shown in (**d**) and (**e**). Representative images from 3 independent experiments are shown. All scale bars=5μm.

Supplementary Figure 7

a

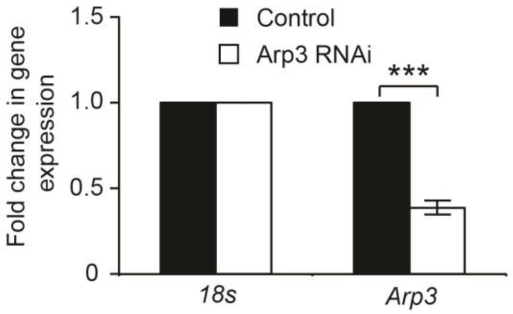

b

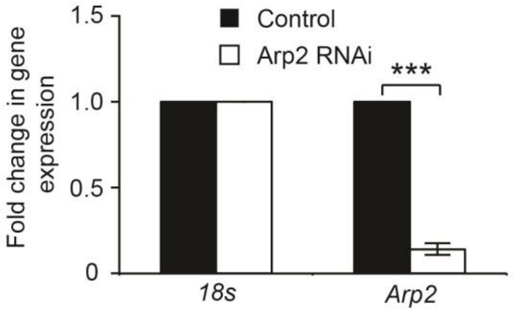

c

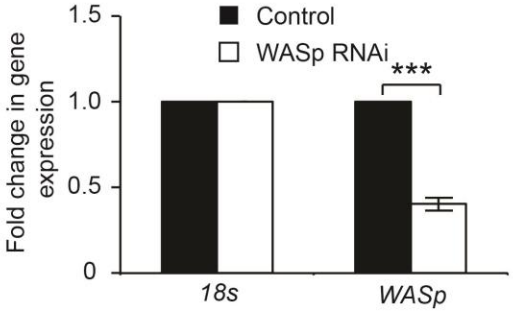

d

**gene**	**primers**
18s rRNA	Sense: 5' - CTTTCTGGGCAAGCAGTTGGAGG - 3'<br>Anti-sense: 5' - GGCACCCAGGAGACGTGC - 3'
Arp2	Sense: 5' - TGATTAAGTTACTGTTACTA - 3'<br>Anti-sense: 5' - GCTTCTCCTTCATTATGC - 3'
Arp3	Sense: 5' - ACTACTTCCTGCTGACTGAG - 3'<br>Anti-sense: 5' - CCTGGACGGCGATATACA - 3'
WASp	Sense: 5' - TCATCTACGACTTCATACAG - 3'<br>Anti-sense: 5' - GTTCTTTGGTTGCCATTAG - 3'

Supplementary Figure 7. In vivo RNAi results in gene knockdown. Quantitative real time PCR to quantitative transcript levels of (a) *Arp3*, (b) *Arp2* or (c) *WASp* following in vivo RNAi. RNA levels were normalized to 18S. Values represent mean values ± S.E.M. from four experiments. Student's *t*-test was used to calculate *P*-values. ***P<0.001 (d) Primer pairs used for quantitative real time PCR.

Supplementary Figure 8

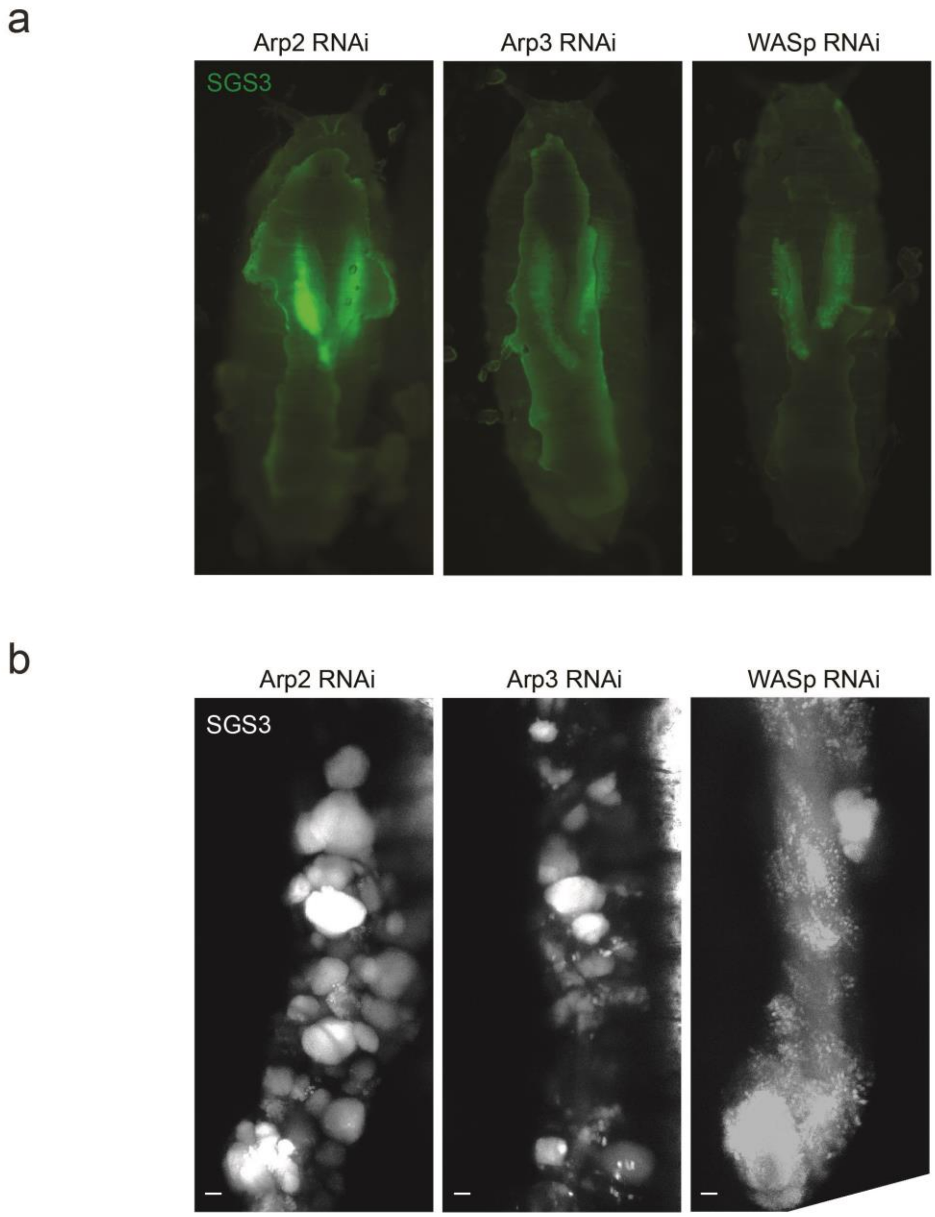

Supplementary Figure 8. Arp2/3 and WASp are required for in vivo cargo secretion (a) In vivo imaging of SGs in prepupae expressing Sgs3-GFP (green) and RNAi to *Arp3, Arp2 or WASp*. Sgs3-GFP is not efficiently excreted by the animal. Instead, Sgs3-GFP remains within the SGs. (b) In vivo confocal imaging of SGs shown in (a) shows large Sgs3-GFP (white) aggregates within individual secretory cells. Representative maximum projections of confocal Z-stacks from 2 independent experiments are shown. All scale bars=20µm.
